# Placental magnetic resonance imaging: normal appearance, anatomical variations, and pathological findings

**DOI:** 10.1590/0100-3984.2020.0010

**Published:** 2021

**Authors:** Maria Inês Novis, Ana Paula Carvalhal Moura, Agnes de Paula Fernandes Watanabe, Luciana Carla Longo e Pereira, Gisele Warmbrand, Giuseppe D'Ippolito

**Affiliations:** 1 Fleury Medicina e Saúde, São Paulo, SP, Brazil.; 2 Escola Paulista de Medicina da Universidade Federal de São Paulo (EPM-Unifesp), São Paulo, SP, Brazil.

**Keywords:** Magnetic resonance imaging, Placenta/diagnostic imaging, Placenta accreta/diagnosis, Ressonância magnética, Placenta/diagnóstico por imagem, Placenta acreta/diagnóstico

## Abstract

Placental magnetic resonance imaging (MRI) has been increasingly requested, especially for the evaluation of suspected cases of placental adhesive disorders, generally known as placenta accreta. Abdominal radiologists need to become familiar with normal placental anatomy, anatomical variations, the current terminology, and major placental diseases that, although rare, are important causes of maternal and fetal morbidity and mortality. The aim of this didactic pictorial essay is to illustrate various findings on placental MRI, as well as to emphasize the importance of communication between radiologists and obstetricians in the search for best practices in the management of the affected patients.

## Abstract (pt)

A ressonância magnética placentária tem sido cada vez mais solicitada, sobretudo na avaliação de casos suspeitos de acretismo. Os radiologistas abdominais precisam se familiarizar com a anatomia placentária normal, variações anatômicas, terminologias atuais e principais doenças placentárias que, embora raras, são causas importantes de morbimortalidade materno-fetal. O objetivo deste ensaio é ilustrar, de maneira didática, diferentes achados placentários e enfatizar a importância da comunicação entre radiologistas e obstetras na busca da melhor conduta para as pacientes.

## INTRODUCTION

Analysis of the placenta is part of the routine prenatal evaluation, initially performed by Doppler ultrasound. For situations in which the ultrasound findings are inconclusive, magnetic resonance imaging (MRI) is employed, because it presents good tissue resolution, regardless of maternal biotype or fetal position, and allows the acquisition of multiplanar images without exposing the patient to ionizing radiation^(1)^.

The growing number of surgical procedures involving manipulation of the uterus has led to a greater number of cases in which there is suspicion of a placental accreta spectrum (PAS) disorder, with a consequent increase in requests for placental MRI^(2)^.

Radiologists need to become familiar with placental MRI, because placental disorders, although rare, have the potential to cause significant maternal and fetal morbidity and mortality. The objective of this pictorial essay is to review the normal placental anatomy, anatomical variations, the main disorders, and the current terminology, using didactic examples.

## NORMAL PLACENTAL ANATOMY ON MRI

Placental MRI examinations should be performed in 1.5-T or 3.0-T scanners, with a phased-array coil, ideally between 28 and 32 weeks of gestation. The patient should be in the supine position, with a minimally to moderately full bladder. The use of gadolinium should be avoided, because intrauterine exposure to gadolinium has been associated with an increased incidence of stillbirth and neonatal death, such exposure also being associated with an increased risk of infiltrative skin conditions, as well as rheumatological and inflammatory diseases in children^(3)^. Our suggested protocol can be performed in 20-30 min and is summarized in Table 1.

**Table 1:** Summary of the proposed protocol.

Sequence	Plane	Thickness	Objective
T2 FSE	Coronal, saggital, axial	≤ 4 mm	Placental localization, study of the cervix, identification of signs of PAS disorder
T2 FIESTA fat-sat	Coronal, saggital, axial	4 mm	Study of the anatomy, margins, and vascularization of the placenta
T1 3D fat-sat	Saggital	3 mm	Investigation of bleeding and hemorrhage (intraplacental or extraplacental)
T2 FSE (uterine cervix)	Saggital	4 mm	Measurement of the distance between the placental margin and the internal cervical os
DWI (b: 0/50 and 600/1000)	Axial	≤ 5 mm	Identification of placental invasion of in adjacent structures

**Figure 1:**
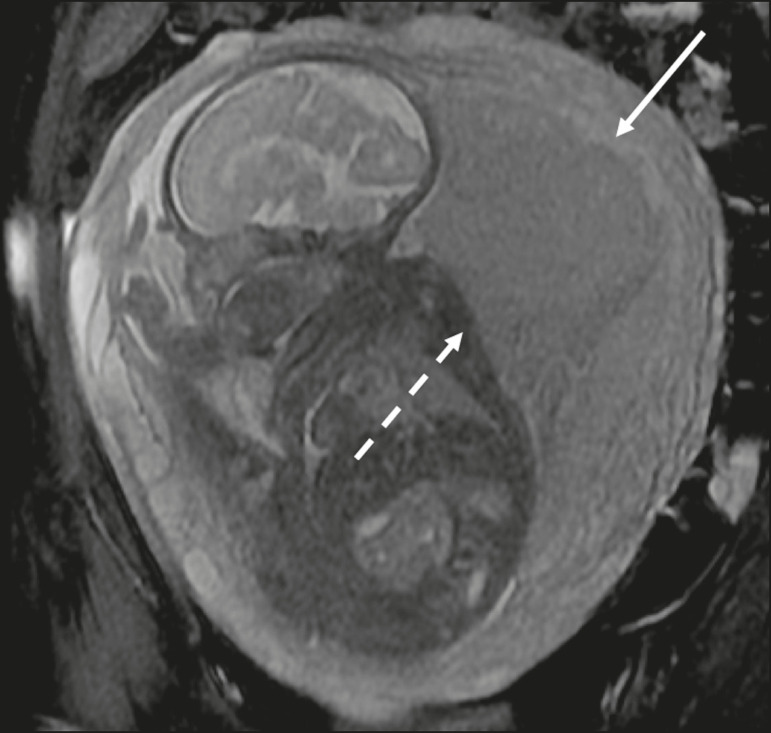
Coronal fat-sat FIESTA T2WI of a gravid uterus, with an inverted pear shape, the placenta on the left side of the uterus body showing a maternal surface (solid arrow) and a fetal surface (dashed arrow).

**Figure 2:**
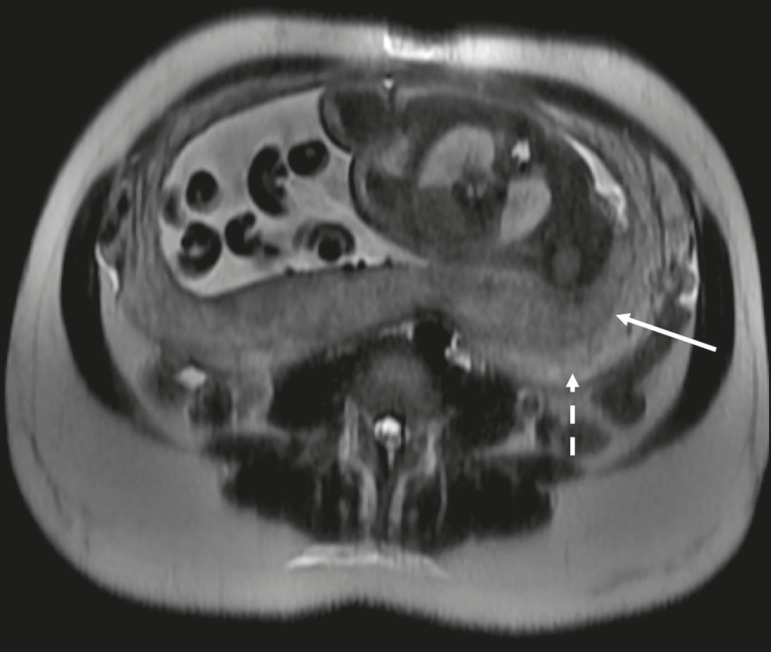
Axial T2WI acquired at 26 weeks of gestation showing a posterior placenta, with clear placenta-myometrium interface (solid arrow), and a three-layered myometrium with a hyperintense central signal on T2WI (dashed arrow).

**Figure 3:**
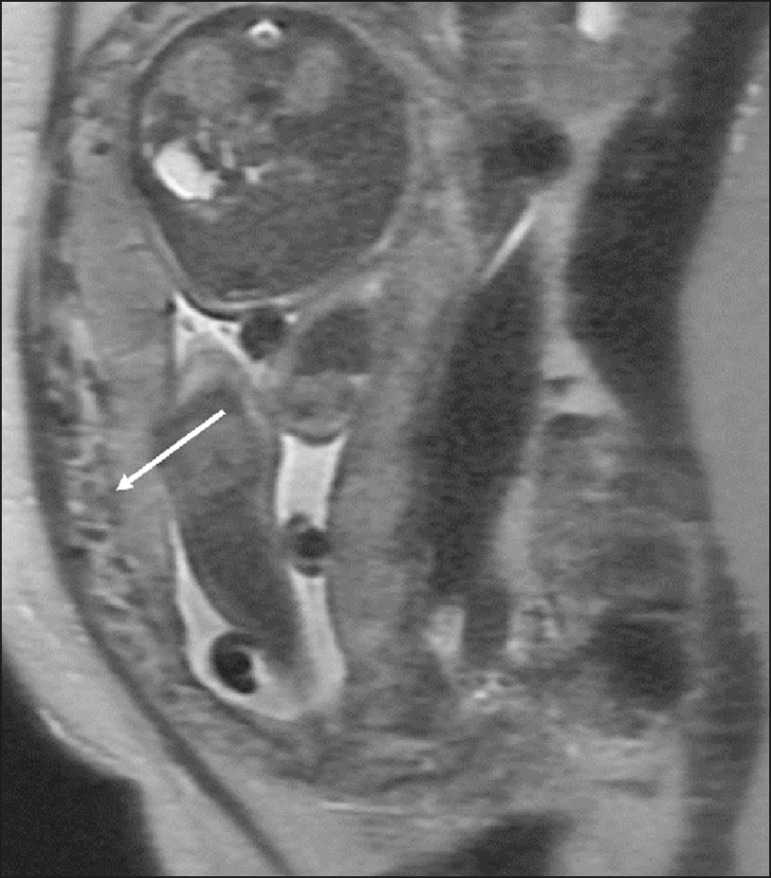
Sagittal T2WI acquired at 33 weeks of gestation showing myometrial tapering and retroplacental flow voids (arrow).

The gravid uterus has smooth contours and an inverted pear shape, the body and fundus being wider than the lower segment^(2)^, as illustrated in Figure 1. The placenta has a fetal surface (the chorionic plate) and a maternal surface (the basal plate), the latter being adjacent to the retroplacental clear space (Figure 1). In the second trimester of pregnancy, the placenta has a flat, discoid morphology, with a smooth surface and homogeneous signal intensity on MRI, which allows good visualization of the placenta-myometrium interface, and the myometrium has a triple-layered aspect with a hyperintense central signal on T2-weighted imaging (T2WI), as shown in Figure 2. With placental maturation in the third trimester, the cotyledons come to be better defined (especially in 3.0 T scanners) and there is progressive myometrial tapering, lobulation of the fetal surface, and subplacental vascularity, characterized by flow voids^(2)^, as shown in Figure 3.

The thickness of the placenta increases with gestational age and must be measured in its middle portion, close to the insertion of the umbilical cord and perpendicular to the placental long axis, with a normal thickness of 2-4 cm. Hematological and systemic vascular diseases with microinfarcts are associated with placental thinning, whereas hydrops fetalis, prenatal infections, diabetes, and maternal anemia are classically associated with placental thickening^(2,4)^.

## ANATOMICAL VARIATIONS

Placental anatomical variations comprise the following^(1,2,4)^:

- Succenturiate lobe-a smaller accessory lobe, which increases the risk of placental retention (Figure 4A)
  **Figure 4:**
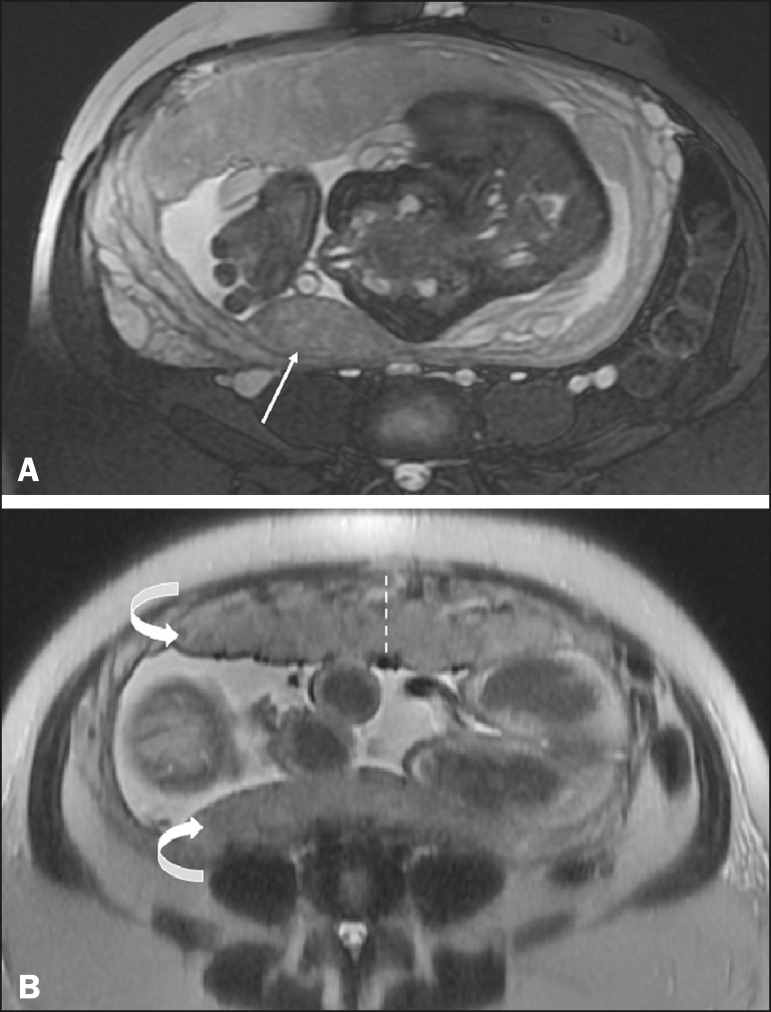
A: Axial fat-sat T2WI showing an anterior placenta with a posterior succenturiate lobe, smaller (arrow). B: Axial T2WI showing a bilobed placenta (curved arrows), with the umbilical cord inserting into the main placenta. Measurement of placental thickness should be performed at the level of the umbilical cord insertion, perpendicular to the long placental axis (dashed line).
- Bilobed placenta-an accessory lobe of similar dimensions, with insertion of the umbilical cord in the main placenta, which increases the risk of vasa previa and rupture of the vessels between the lobes during labor (Figure 4B)
- Circumvallate placenta-disproportion between the placental surfaces, the chorionic plate being smaller, with spare peripheral placental tissue, increasing the risk of placental detachment, prematurity, low birth weight, and chronic lung disease of infancy
- Placenta membranacea and diffuse placenta-a rare condition in which the placenta circumferentially occupies the entire periphery of the chorion, predisposing to placenta previa (PP), vaginal bleeding, and premature delivery

The placental insertion of the umbilical cord can vary, as follows^(2,4-6)^:

- Typical-central, in the middle third of the placenta (Figure 5A).
  **Figure 5:**
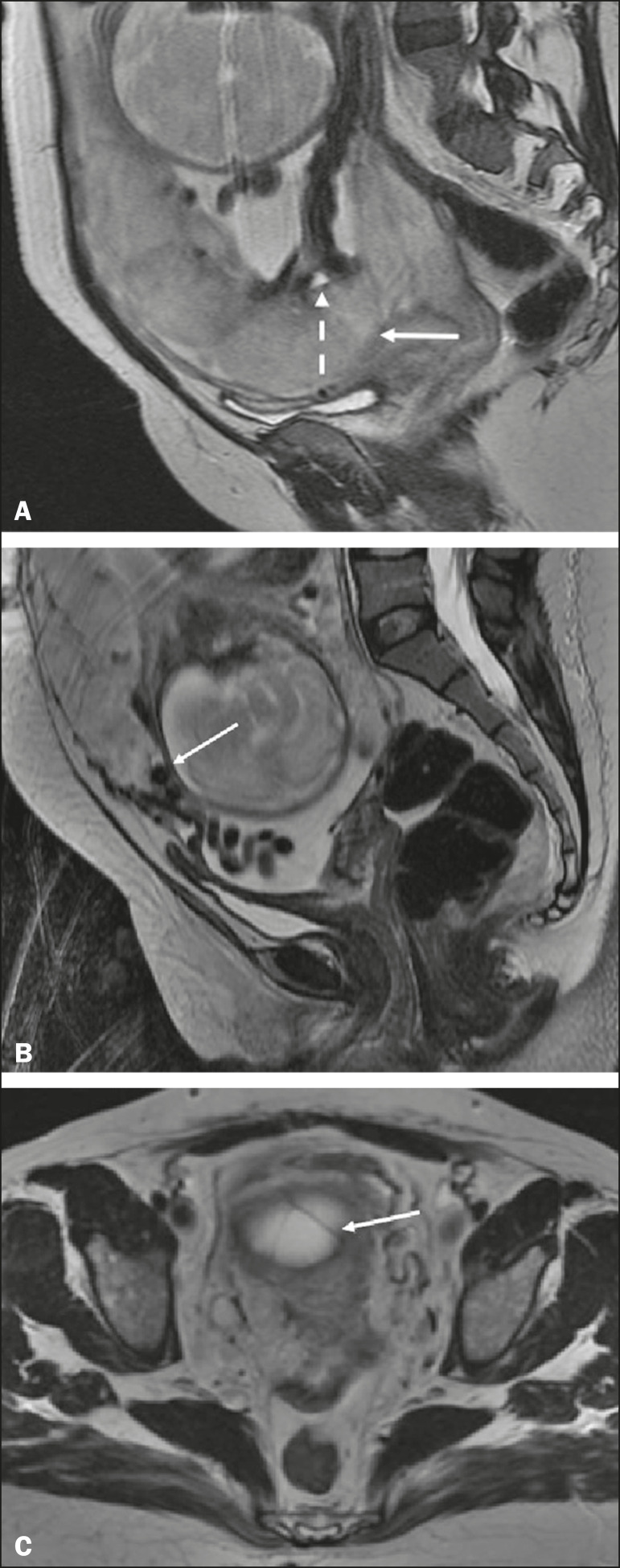
A: Sagittal FSE T2WI of the uterine cervix. PP centered in the region of the ICO (solid arrow) covering it completely. Umbilical cord inserted in the middle third of the placenta (dashed arrow). B: Sagittal FSE T2WI showing an anterior placenta with marginal insertion of the umbilical cord, less than 0.5 cm from the placental margin (arrow). C: Axial FSE T2WI showing vasa previa with thin vessels crossing the ICO (arrow).
- Marginal-located 1-2 cm from the placental margin (prevalence of 7-9% in a single pregnancy and 24-33% in twin pregnancies); when the distance from the placental margin is ≤ 0.5 cm, there is a risk of progression to velamentous insertion, and it is important to include this measure in the radiology report (Figure 5B)
- Velamentous-the vessels leave the placenta, running between the chorion and the amnion, and the umbilical cord is inserted in the membranes (prevalence of 1% in singleton pregnancies and 15% in monochorionic twin pregnancies); velamentous cord insertion is associated with low birth weight and abnormal fetal heartbeat at birth

Vasa previa is a condition related to velamentous cord insertion or to a bilobed placenta/succenturiate lobe, in which thin vessels run through the membranes close to the internal cervical os (ICO) and below the fetal presentation (prevalence of 0.04%), increasing the risk of membrane rupture, bleeding, and fetal exsanguination; cesarean section is indicated (Figure 5C).

## LOW-LYING PLACENTA VERSUS PP

**Figure 6:**
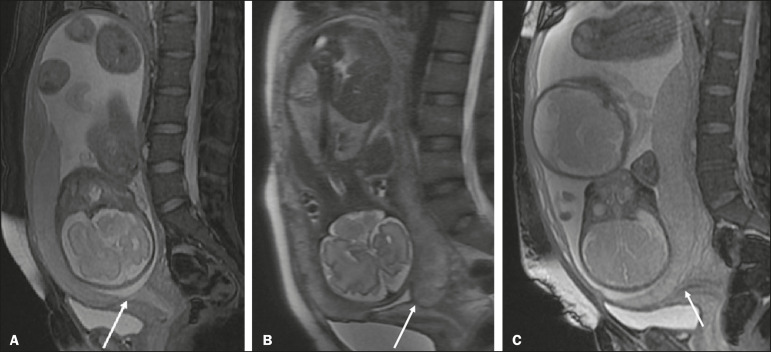
Sagittal fat-sat FIESTA T2WI sequences. A: Low-lying placenta, the placental margin in contact with the ICO (arrow), without covering it. B: Posterior PP, in which the placental margin partially covers the ICO (arrow). C: Posterior PP, in which the placental margin totally covers the ICO (arrow).

A low-lying placenta is one that is implanted in the inferior uterine segment, with a margin less than 2 cm from the ICO without covering it, and is best evaluated in the sagittal plane of the cervix (Figure 6A). A low-lying placenta is associated with a higher risk of bleeding^(5)^.

The term PP is reserved for cases where the placenta partially or completely covers the ICO (Figures 6B and 6C). The radiologist should include this information in the report in a descriptive manner, avoiding the terms marginal, partial, complete, and central (total) PP. The diagnosis of PP should be made only in the third trimester (around 30 weeks), given that placental migration can still occur prior to that time^(4,6)^. The main risk factors for PP are as follows^(5)^: anterior cesarean section or uterine scarring; advanced maternal age; multiparity; curettage; twinning; a history of placenta previa (recurrence in 4-8%); smoking; and the use of illicit drugs, such as cocaine.

## PLACENTAL ABRUPTION

**Figure 7:**
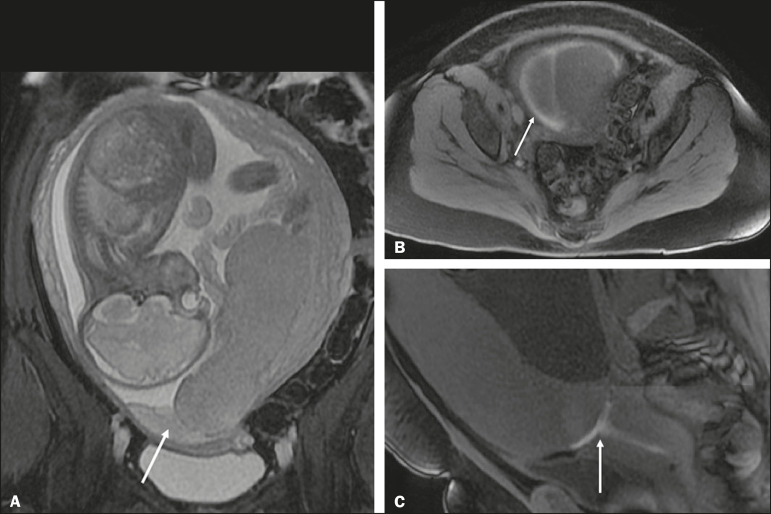
Fat-sat FIESTA sequences. Coronal T2WI (A) and axial T1WI (B) showing PP with retroplacental hematoma, with an intermediate signal on T2WI (arrow) and a hyperintense signal on T1WI (arrow). C: Sagittal fat-sat T1WI showing a PP with laminar blood collections in the retroplacental space and in the cervical canal (arrow).

Placental abruption is a rare condition (occurring in < 1% of pregnancies) that causes premature birth and increased fetal mortality. Ultrasound can produce a false-negative result in more than 50% of cases, because the hemorrhage can be isoechoic to the placenta. On MRI, the hemorrhage is quite different from the placenta, it may show restricted diffusion on diffusion-weighted imaging (DWI), and the intensity of its signal can help differentiate between the acute, subacute, and chronic phases, thus facilitating the prediction of the stability of the bleed^(2,4)^. Placental abruption is categorized as follows^(1,2)^: subchorionic (preplacental, between the membranes and the placenta, due to rupture of uteroplacental veins, accounting for approximatyelly 57% of cases); retroplacental (due to rupture of small deciduous arteries, accounting for 43% of cases; Figure 7); placental; and subamniotic (between the amnion and the chorion, rarely seen).

## PAS DISORDERS

The PAS disorders result from excessive trophoblastic invasion and anomalous decidualization. The incidence of PAS disorders has been increasing and is proportional to the number of cesarean sections (risk of 11% for one cesarean section and 61% for three cesarean sections). Other risk factors include previous uterine surgery, PP, assisted reproduction, advanced maternal age, and Asherman's syndrome^(7,8)^. As the name suggests, PAS disorders encompass a spectrum of diseases, from placenta accreta, in which there is direct contact between the placenta and the myometrium (partial invasion), to placenta percreta, in which there is total myometrial invasion, and placenta increta, which is characterized by extension of the placenta beyond the uterine serosa and, in some cases, invasion of adjacent organs, such as the bladder. Differentiating among those forms can be a challenge for radiologists^(4,8)^.

**Figure 8:**
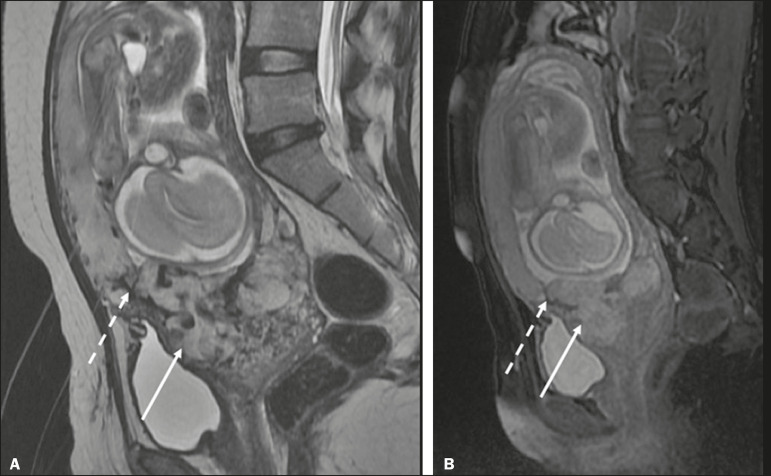
A 35-year-old patient at 22 weeks of gestation with a history of one cesarean delivery. A: Sagittal FSE T2WI showing a PP with uterine bulge, placental protrusion (solid arrow), heterogeneity, and dark intraplacental bands with placental retraction (dashed arrow), loss of the uterine-placental interface and myometrial thinning suggesting PAS disorder (ideal sequence for assessing accretion). B: Sagittal fat-sat FIESTA T2WI showing the same signs, less clearly than in the FSE sequence. Placenta accreta was confirmed at delivery, and total hysterectomy was performed.

**Figure 9:**
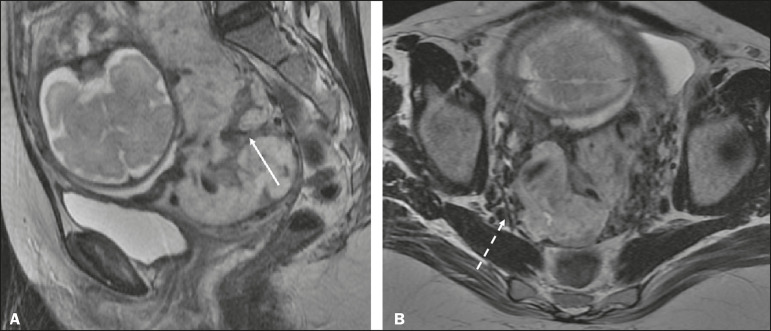
A 37-year-old patient at 32 weeks of gestation, with a history of one normal delivery and two abortions. Sagittal FSE T2WI (A) and axial FSE T2WI (B) showing placental protuberance and heterogeneity, dark intraplacental bands (solid arrow), and increased subserous vascularity (dashed arrow). Placenta accreta was confirmed at delivery, and total hysterectomy was performed.

The suspicion of a PAS disorder is the main indication for MRI, and prenatal diagnosis is extremely important because it allows better planning with a multidisciplinary team and possible uterine artery embolization, thus avoiding hysterectomy^(8)^. On MRI, PAS disorder abnormalities can be divided into major and minor signs, with diagnostic specificity ≥ 80% and < 80%, respectively. The major signs comprise abnormal uterine bulge; placental protrusion; placental heterogeneity; bands with a hypointense signal (dark intraplacental bands) on T2WI, accompanied by placental retraction; and placental protrusion into adjacent structures (including the ICO). The minor signs include a dark hypointense band (> 2.0 cm in length and > 1.0 cm in thickness) on T2WI; irregular placenta-myometrium interface; myometrial thinning; abnormal placental vascularity; and subserous hypervascularity (Figures 8 and 9). It is noteworthy that the ideal sequence for evaluating these signs is a T2-weighted fast spin-echo (FSE) sequence, which provides the best characterization of placental heterogeneity^(1,2,4,7,9)^.

## GESTATIONAL TROPHOBLASTIC DISEASE

Gestational trophoblastic disease (GTD) is a relatively uncommon disease, with a spectrum of benign and malignant presentations, ranging from partial or complete hydatidiform mole to persistent trophoblastic neoplasia, including invasive mole, choriocarcinoma, and placental site trophoblastic tumor. The main risk factors are previous trophoblastic disease and advanced maternal age. It is accompanied by an increase in beta-human chorionic gonadotropin level, hyperemesis, uterine enlargement disproportionate to gestational age, bleeding, and enlarged ovaries with corpus luteum cysts^(1)^.

**Figure 10:**
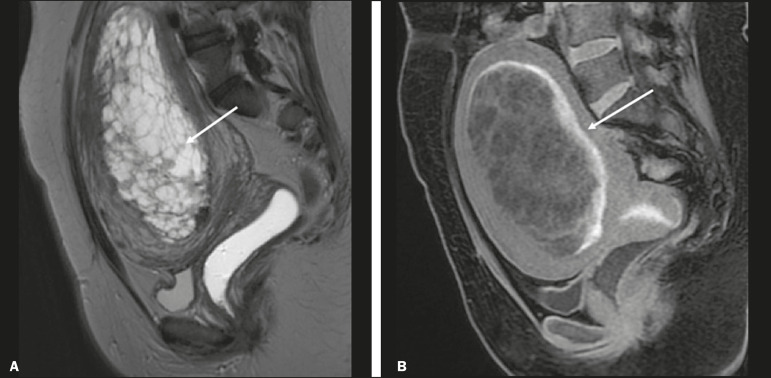
A: Sagittal FSE T2WI showing the uterine cavity distended by heterogeneous content with multiple cysts (arrow). B: Sagittal fat-sat T1WI showing laminar blood surrounding a cyst and extending to the cervical canal (arrow). Post-hysterectomy pathology finding: complete mole.

A complete hydatidiform mole, without an embryo, is the most common form of GTD. Persistent trophoblastic neoplasia has an incidence of up to 29% after the occurrence of a mole, and MRI is useful in the evaluation of myometrial invasion and local staging^(2)^, as illustrated in Figure 10.

Chorioangioma-located near the insertion of the umbilical cord-is the most common nontrophoblastic placental tumor (occurring in 1% of placentas), followed by teratoma and metastases, such as those originating from a melanoma or lung neoplasm, as well as leukemic infiltration^(1,2,4)^.

## RETAINED PRODUCTS OF CONCEPTION

Suspicion of retained products of conception arises when there is incomplete expulsion of the placenta or persistent vaginal bleeding after delivery or abortion, and the diagnosis is typically confirmed by ultrasound. On MRI, it appears as an eccentric, heterogeneous, hypervascularized or hypovascularized intracavitary mass-usually with a signal that is hyperintense on T1WI and hypointense or intermediate on T2WI-with obliteration of the junctional zone, myometrial thinning, and intracavitary blood. The differential diagnosis includes GTD and acquired uterine arteriovenous malformation^(1,10)^.

## FURTHER CONSIDERATIONS

The evaluation of placental MRI should also include the length of the uterine cervix, in a descriptive manner, terms such as "short cervix" being avoided. In examinations performed at 18-24 weeks of gestation, a cervical length < 3 cm (especially if < 2 cm) can help define the risk of premature birth. After 24 weeks of gestation, obstetricians should interpret this measure in correlation with previous examinations and the clinical profile^(5)^. It is also important to describe any additional findings, such as uterine fibroids, ovarian masses, gross fetal abnormalities, oligohydramnios, and polyhydramnios.

Placental MRI has limitations inherent to the patient, such as uterine contractions, fetal/maternal movements, claustrophobia, and difficulty in lying down in the third trimester, or to the method, such as the relatively long examination time, high cost, and need for radiologists familiar with placental disorders. In the past, examinations were intended only to determine the location of the placenta. Currently, placental imaging is believed to be vital for understanding placental physiology and efficiency, with a likely future contribution to understanding premature births, fetal growth restriction, and pre-eclampsia. The possibility of performing three-dimensional imaging and functional MRI for the evaluation of placental vascularity, together with greater use of DWI and blood oxygen level-dependent imaging, as well as quantitative (radiomic) texture analysis of placental MRI, may add information in the near future^(4)^.

## CONCLUSION

Placental MRI has been used as an important complement to ultrasound, and it is essential that radiologists are familiar with the main aspects of the normal and abnormal placenta. Communication between radiologists and obstetricians is crucial for the definition of best practices, with the objective of reducing maternal and fetal morbidity and mortality.
