# ERRATUM

**DOI:** 10.1590/0100-3984.2021.er-1

**Published:** 2021

In the article *Placental magnetic resonance imaging: normal appearance, anatomical variations, and pathological findings* (DOI: http://dx.doi.org/10.1590/0100-3984.2020.0010), published in **Radiologia Brasileira**, 54(2):123-129, the passage

...to placenta “percreta”, in which there is total myometrial invasion, and placenta “increta”...

on page 126, paragraph 6, lines 11 and 12,

should read:

...to placenta “increta”, in which there is total myometrial invasion, and placenta “percreta”...

Prof. Dr. Edson Marchiori

Editor in Chief of **Radiologia Brasileira**

